# A 3D-microtissue-based phenotypic screening of radiation resistant tumor cells with synchronized chemotherapeutic treatment

**DOI:** 10.1186/s12885-015-1481-9

**Published:** 2015-06-10

**Authors:** Nataša Anastasov, Ines Höfig, Vanja Radulović, Simon Ströbel, Michael Salomon, Jan Lichtenberg, Ina Rothenaigner, Kamyar Hadian, Jens M. Kelm, Christian Thirion, Michael J. Atkinson

**Affiliations:** 1 Institute of Radiation Biology, Helmholtz Zentrum München - German Research Center for Environmental Health, Ingolstaedter Landstr. 1, 85764 Neuherberg, Germany; 2 Insphero AG, Schlieren, Switzerland; 3 Sirion Biotech GmbH, Martinsried, Germany; 4 Assay Development and Screening Platform, Institute of molecular Toxicology and Pharmacology, Helmholtz Zentrum München - German Research Center for Environmental Health, Neuherberg, Germany; 5 Chair of Radiation Biology, Technical University of Munich, Munich, Germany

**Keywords:** Radiation therapy, Potentiating drugs, High content screen (HCS), 3D-microtissues, Tumor growth, Vinblastine

## Abstract

**Background:**

Radiation resistance presents a challenge to the effective treatment of cancer. If therapeutic compounds were capable of resensitizing resistant tumours then a concurrent chemo-radiation treatment could be used to overcome radiation resistance.

**Methods:**

We have developed a phenotypic assay to investigate the response of radiation resistant breast cancer cells grown in 3D-microtissue spheroids to combinations of radiation and established chemotherapeutic drugs. The effects were quantified by real time high content imaging of GFP detection area over 14 days. Ten established chemotherapeutic drugs were tested for their ability to enhance the effects of radiation.

**Results:**

Of ten analysed chemotherapeutics, vinblastine was the most effective compound, with docetaxel and doxorubicine being less effective in combination with radiation. To investigate the response in a model closer to the in vivo situation we investigated the response of heterotypic 3D microtissues containing both fibroblasts and breast cancer cells. Drug treatment of these heterotypic 3D cultures confirmed treatment with radiation plus vinblastine to be additive in causing breast cancer growth inhibition. We have validated the screen by comparing radiation sensitizing effects of known chemotherapeutic agents. In both monotypic and heterotypic models the concurrent treatment of vinblastine and radiation proved more effective inhibitors of mammary cancer cell growth. The effective concentration range of both vinblastine and radiation are within the range used in treatment, suggesting the 3D model will offer a highly relevant screen for novel compounds.

**Conclusions:**

For the first time comfortable 3D cell-based phenotypic assay is available, that allows high throughput screening of compounds with radiation therapy modulating capacity, opening the field to drug discovery.

**Electronic supplementary material:**

The online version of this article (doi:10.1186/s12885-015-1481-9) contains supplementary material, which is available to authorized users.

## Background

The rapid evolution of resistance to both conventional and small molecule therapies is a challenging problem in oncology. One approach to overcome resistance is to use combinatorial treatments that exploit their synergies. The combination of chemotherapy and radiation treatment is emerging as a potentially effective combinatorial regimen, although the optimal mix has not been identified [1, 2].

A major drawback in identifying potentially radiation-sensitizing chemotherapeutic agents is the lack of high throughput screening (HTS) vehicles to identify possibly beneficial combinations. These are needed to replace conventional clonogenic survival assays of radiation treatment as these are too expensive and time consuming to operate in a first-pass screening mode. Moreover, there are growing concerns that monolayer and monotypic (2D) cellular screening assays may not effectively reproduce the response of a three-dimensional (3D) solid tumor to pharmacological compounds [3–5].

Multicellular 3D spheroid models have been proven to be representative of in vivo tumors [6–8]. However, classical 3D technologies such as tumor spheroid analysis using hanging drops, microencapsulation, and liquid overlays are laborious and not sufficiently reproducible for use as high throughput screens [9–12]. We have modified an existing hanging drop 3D-microtissue technology (Insphero, AG) to develop a high content screen to interrogate potential radiation sensitizing compounds. Major advantage of such new screen technology is single spheroid growth analysis (per well) after chemo- or radiation treatment. In validating the assay we examined a panel of ten standard chemotherapeutic compounds for their ability to potentiate the anti-tumour action of radiation against the radiation resistant T47D mammary cancer cell line.

Co-culturing cancer cells with fibroblasts in 3D heterotypic microtissues can mimic breast cancer heterogeneity, allowing a more physiological response to screening [13–15]. To fully recapitulate the complexity of breast cancer we established heterotypic cultures of normal human dermal fibroblasts (NHDF) and a panel of three mammary cancer cell lines (T47D, MDA-MB-361 and MDA-MB-231). Here we report the identification of vinblastine as a potential radiosensitizing treatment in both monotypic and heterotypic 3D-microtissues.

## Methods

### Growth and maintenance of cell lines

The T47D breast cancer cell line (HTB-133), the MDA-MB-361 (HTB-27) and the MDA-MB-231 (HTB-26) cell lines were a kind gift from Professor M. Aubele, Institute of Pathology, Helmholtz Center Munich. The T47D breast cancer cell line and the GFP/RFP lentivirus modified T47D-GFP and T47D-RFP cell lines were maintained in RPMI 1640 (Roswell Park Memorial Institute) medium supplemented with 10 % FCS and human insulin (10 μg/ml). MDA-MB-231 cell line and the GFP/RFP expressing MDA-MB-231-GFP and MDA-MB-231-RFP cell lines were maintained in DMEM (Dulbecco’s Modified Eagle Medium) supplemented with 10 % FSC and non-essential amino acids (Sigma Aldrich, USA). MDA-MB-361 cell line and the GFP/RFP expressing (MDA-MB-361-GFP and MDA-MB-361-RFP) cell lines were maintained in DMEM supplemented with 20 % FCS. Primary normal human dermal fibroblasts (NHDF) expressing GFP were temperature sensitive immortalized by protocols from Sirion Biotech GmbH (GE) and were maintained in Fibroblast growth medium (Promocell, GE) supplemented with 0.4 mg/ml G418. Additionally all GFP/RFP expressing cell lines were supplemented with 0.3 μg/ml Puromycin (Sigma Aldrich, USA) for stable cell selection of fluorescent marker expression. The human embryonic kidney HEK293T (DSMZ, Germany) cells were used for lentivirus productions and grown in DMEM medium with 10 % FCS. Cultivation was performed under standard conditions in water humified 37 °C incubator with 5 % CO_2_, either for 2D or 3D cell analysis. Cell lines were checked for mycoplasma contamination using the MycoAlert Detection Kit (Lonza Group Ltd, CH) and their identity verified by genetic profiling using the PowerPlex® 16 System (Eurofins/MWG Operon, GE). Research involving human patient material and data with ethics committee approval was not used for this study.

### Lentivirus production and infection of breast cancer cell lines

Replication-defective lentiviral particles were produced by transient co-transfection of HEK293T cells in a 10 cm petri dish using Lipofectamine 2000 (Life Technologies, USA) according to the manufacturer’s instructions. Transfection mix contained 16 μg, 8 μg and 4 μg of packaging plasmids pMDLg/pRRE, pRSV.Rev and pMD2.G (a kind gift from D. Trono, École polytechnique fédérale de Lausanne, CH) and 8 μg of lentiviral transduction vector pGreenPuro (pGP) expressing copGFP (System Biosciences, USA). The virus particles were harvested 48 hours after transfection, cleared and concentrated as described [16]. According to virus titer determination virus productions ranged between 10^8^ and 10^9^ TU/ml and viral infection of T47D breast cancer cells was performed using previously described protocols [16–19]. Briefly, 2 × 10^5^ cells per well were infected with 4 × 10^5^ TU/ml (2 MOI) and three days after infection GFP expression was monitored. Correspondingly the T47D, MDA-MB-361 and MDA-MB-231 breast cancer cells were stable transduced with red fluorescence protein (RFP) lentiviral expression vectors using protocols from Sirion Biotech GmbH (GE) and maintained with 0.3 μg/ml puromycine [20]. Stable GFP or RFP labeled cells were seeded in Gravity PLUS™ plates (InSphero AG, CH) and treated as described below.

### Generation of monotypic tumour 3D-microtissues and radiation treatment

**Fig. 1:**
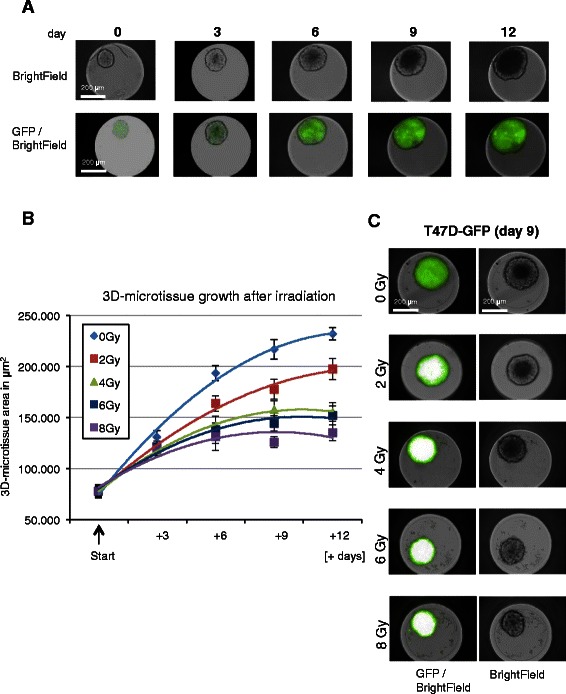
Growth analysis of 3D-microtissues with constitutive lentiviral-GFP expression. **a** Example of Operetta bright-filed and GFP detection in breast cancer 3D-microtissue spheroids after indicated time points (days after seeding in assay plates). **b** 3D-microtissue growth analysis after radiation (GFP plot in μm^2^) – area quantification of spheroid growth delay after irradiation at indicated time points and different radiation doses. Data are averages ± SD (*n* = 3). **c** Example of Operetta bright-filed and GFP area detection 9 days after radiation treatment. Operetta laser scanning instrument operates with constant excitation times (1 ms), nevertheless fluorophore saturation was detected after radiation treatment, confirming that absolute fluorescence intensity cannot be used for quantification

Cell density in media was estimated using a hemocytometer prior seeding the cells in (96-well) 3D hanging drop culture plates. 3D microtissues were generated ranging from 200 to 2000 cells per well and breast cancer 3D microtissues started with 500 cells per well were chosen as starting point showing adequate growth kinetics and low interwell variations (bellow 10 %) for the following studies. Standard methods describe production of spheroids using 10^6^ cells that are plated in 100-mm pre-coted Petri dishes to develop mammary spheroids (within 6 to 9 days) raging in size between 250 μm and 350 μm [7]. In our study 3D-microtissues were formed by seeding T47D, MDA-MB-361 and MDA-MB-231 cells into the Gravity PLUS™ 96 well plate (500 cells/well) and maturing them for 3 days in hanging drops, followed by transfer of the single spheroids into the Gravity TRAP™ (receiver) assay plates (InSphero AG, CH). After one day of recovery (defined as day 0 of treatment), tissues were sham irradiated (0 Gy) or irradiated with a single acute dose of 2 Gy, 4 Gy, 6 Gy or 8 Gy with a Cs-137 irradiator (HWM D-2000, Siemens, GE) delivered a dose rate of 0.5 Gy/min. The exposed and sham irradiated 3D-microtissues were subsequently incubated at 37 °C with 5 % CO_2_ for indicated time points. The experiment was repeated for each dose in quadruplicates (*n* = 4). The 3D-microtissues (spheroids) were treated with different single doses of radiation from 2 Gy to 8 Gy and subsequently growth was analysed every 3 days after treatment mostly dependent on experimental set-up and working days (Fig. 1 and Additional file 1: Figure S1). 3D-microtissues from 8 wells were used for cell number counts by hemocytometer. The GFP image-based area (μm^2^) measurement (Additional file 1: Figure S1a) correlates with increased cell number counts per spheroid, confirming efficient 3D-microtissue growth quantification using green microtissue area determination (Additional file 1: Figure S1b). Growth of 3D-microtissues was followed in assay plates for 20 days with Operetta (Perkin Elmer, USA) measurements without additional pipetting steps during assay analysis, except medium change after 6 days and 12 days of analysis (Additional file 1: Figure S1c). Direct quantification of 3D-microtissue fluorescent area (Additional file 1: Figure S1) using a high imaging platform accelerates assay quantification of 3D-microtissue growth after radiation and captures the full range of microtissue phenotypes during analysis.

### 3D-microtissue growth kinetics and treatment with test compounds

3D-microtissue growth was measured for up to 20 days after initiation of treatment (day 0). Complete medium change was performed on day 6 and 12. The 3D-microtissues were treated with different concentrations of chemotherapeutic agents at day 0 concurrent to radiation. DMSO (1 %) was used as control solvent for generating 10 mM stock of Docetaxel, Vinblastine, Actinomycin D, Etoposide, Staurosporine and 5-Fluorouracil. H_2_O was the control solvent for Doxorubicin, Hydrocortisone, Cyclohexamide and 6-Thioguanine (Sigma-Aldrich Co, USA) treatment. Irradiation (2 Gy) was applied once at day 0 and concurrently substances (1 μl/well) were added to 3D-microtissue Gravity-TRAP plates at indicated concentrations.

### Image analysis and 3D-microtissue growth efficiency quantification

Imaging was performed at different time points post-irradiation (over 20 days) using the Operetta® High Content Imaging System (Perkin Elemer, USA). Images from a single plate were acquired in the GFP, RFP and Brightfield channels using the 10xNA objective in wide field mode. Automated quantitative analysis of 3D-microtissue sizes at the different time points was then performed using Harmony®3.1 High Content Imaging and Analysis Software. In the Harmony software, the *Find Image Region* Building Block was then applied to the GFP or RFP channel to detect the microtissues in the well. As a next step, the *Calculate Morphology* Building Block was added to calculate the tissue area (μm^2^) as the final readout. Data generated from 96 wells at different time points were normalized to the starting point (day 0 of irradiation and compound treatment) using control sham irradiated spheroids with 1 % DMSO treatment. Inter-well variations were less than 5 % for monotypic cultures and between 5 and 20 % for heterotypic co-cultures. For statistical analysis the Student’s *t*-test was used.

### Generation of heterotypic 3D-microtissues and combined treatment with compounds and radiation

For the heterotypic 3D-microtissue assays normal human dermal fibroblasts (NHDF) were GFP labelled with lentiviral approach (Sirion Biotech, GE) and co-cultured with RFP breast cancer cell lines (T47D, MDA-MB-361 and MDA-MB-231). NHDF-GFP (1500 cells/well) were mixed with RFP-breast cancer cells (250 cells/well), matured for 3 days in hanging drops, followed by transfer of the heterotypic spheroids into Gravity TRAP™ assay plates (InSphero AG, CH). After 1 day of recovery, microtissues were sham (0 Gy) or with 2 Gy irradiated and concurrently compounds (vinblastine and doxorubicine) were added at 10 nM and 100 nM in quadruplicates to the assay plates. Heterotypic 3D-microtissue growth was measured up to 20 days after initiation of treatment (day 0) and quantified using Operetta High Content Imaging System. A dual laser scan was performed using GFP filter (ex. 460–490 nm and em. 500–550 nm) to measure NHDF-GFP spheroid formation and Alexa-546 Filter (ex. 520–550 nm and em. 560–630 nm) to measure T47D-RFP, MDA-MB-361-RFP and MDA-MB-231-RFP spheroid area formation.

## Results

### 3D-microtissues for high content screening of radiation sensitivity

The growth response of T47D breast cancer cells stably transduced with a lentiviral vector expressing GFP fluorescent protein was followed over 20 days by high content analysis of the (green) microtissue area (Fig. 1). Additional file 1 shows that the area of T47D-GFP spheroids correlated with the change in cell numbers. Treatment with a range of radiation doses (2–8 Gy) induced growth delays that were detectable even at the lowest 2 Gy radiation dose tested (Fig. 1b). Fig. 1c shows representative images of T47D-GFP 3D-microtissues used for GFP area quantification.

**Fig. 2:**
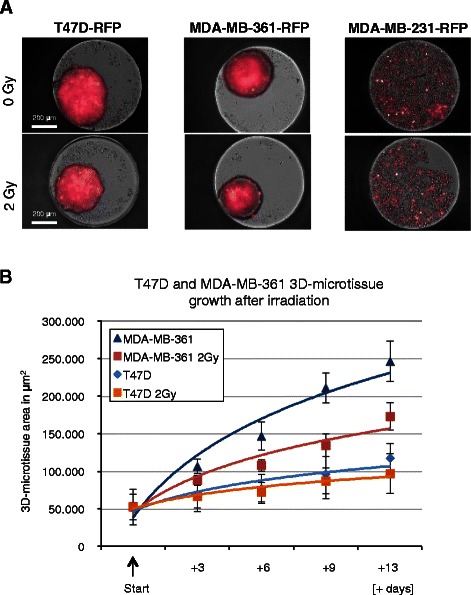
Growth analysis of monotypic 3D-microtissues with constitutive lentiviral-RFP expression. **a** Example of RFP detection for breast cancer 3D-microtissue spheroids generated from T47D-RFP, MDA-MB-361-RFP and MDA-MB-231-RFP cells (9 days after treatment), **b** RFP plot quantification of MDA-MB-361 and T47D spheroid growth delay after 2 Gy irradiation at indicated time points. Data are averages ± SD (*n* = 4). MDA-MB-231 were not quantified using RFP area (μm^2^) settings, as they were not forming consistent spheroid structures during 13 days of analyses

To confirm the radiation effect in other mammary cancer cells 3D-microtissues of MDA-MB-361 and MDA-MB-231 transduced with an RFP expressing lentivirus were evaluated using the hanging drop plates. Figure 2a shows that T47D-RFP and MDA-MB-361-RFP cells readily formed well-packed multi-cellular spheroidal 3D-microtissues, whilst MDA-MB-231-RFP cells lack the capacity to self-aggregate and form microtissues. These latter cells could not be analyzed in 3D monotypic microtissues. A comparison of the growth of T47D-RFP and MDA-MB-361-RFP cells after irradiation confirmed that the assay was able to detect the greater radiation sensitivity of the MDA-MB-361 cells (Fig. 2b).

### Kinetics of the inhibition of 3D-microtissue growth by cytostatic compounds

**Fig. 3:**
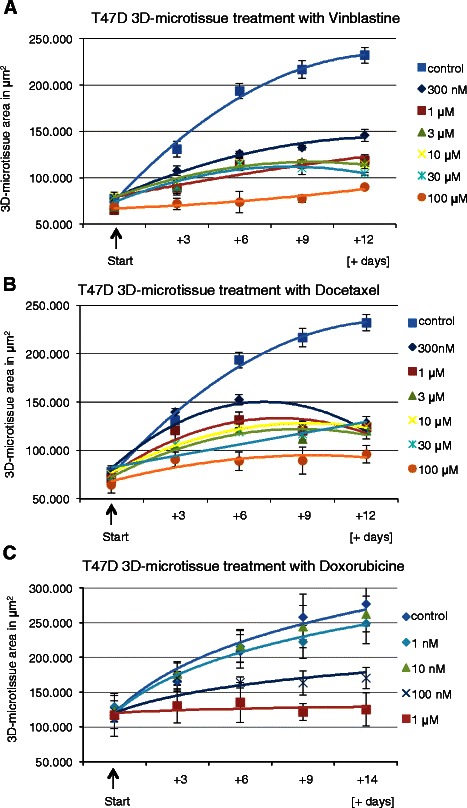
3D-microtissue growth analysis after Vinblastine, Docetaxel and Doxorubicine treatment. GFP plot (area in μm^2^) quantification of spheroid growth delay after **a** Vinblastine, **b** Docetaxel and **c** Doxorubicine treatment at indicated time points and compound concentrations (data are averages ± SD, *n* = 4)

The cytostatic potential of 10 chemotherapeutic compounds was determined using T47D-GFP 3D-microtissues. Results of the three cytostatica most often used in breast cancer treatment (docetaxel, vinblastine and doxorubicine) are presented on Fig. 3. Efficient inhibition of 3D-microtissue growth was detectable with 300 nM of vinblastine (Fig. 3a) and 300 nM of Docetaxel (Fig. 3b). These values correlate well with maximum plasma level concentrations used in anticancer treatment and confirm that the 3D-microtissue assay can provide relevant information on therapeutic potential. The results for the remaining seven compounds are presented in Additional file 2, showing high inhibitory effects for Actinomycin D, Etoposide, Cyclohexamide and 5-FU.

### Combined effects of radiation and chemotherapeutic treatment

**Fig. 4:**
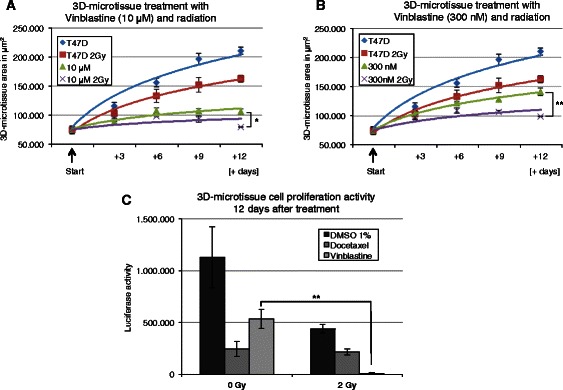
Quantification of radiosensitizing effect after Vinblastine treatment using T47D monotypic 3D-microtissues. GFP plot (area in μm^2^) for control T47D 3D-microtissue and after treatment with 0 Gy and 2 Gy irradiation using (**a**) 10 μM Vinblastine and (**b**) 300 nM Vinblastine at indicated time points. Data are averages ± SD; * indicate statistically (*t*-test) significant changes to corresponding control with Vinblastine treatment at 0 Gy, *p < 0.05; **p < 0.001. (**c**) T47D 3D-microtissues were treated with 0 Gy and 2 Gy radiation including 300 nM Docetaxel or 300 nM Vinblastine. 12 days after treatment 3D-microtissues were lysed directly in assay plates and measured for luciferase activity (Cell-TiterGlo Assay). Data are averages ± SD, * indicate statistically (*t*-test) significant changes to corresponding controls at 0 Gy, **p < 0.001

Vinblastine (Fig. 4), docetaxel and doxorubicine (Additional file 3) were tested in combination with an acute 2 Gy radiation exposure. Combined treatments of docetaxel or doxorubicine with radiation did not produce any increase in efficacy beyond that of the chemotherapeutic compounds alone (Additional file 3 a-d). In contrast, vinblastine treatment combined with irradiation produce an additional inhibitory effect when used in low (300 nM) concentration (Fig. 4b). These results were confirmed using the CellTiter-Glo proliferation assay (Fig. 4c).

### Development of heterotypic 3D-microtissues for combined radiation and chemotherapeutics screening

**Fig. 5:**
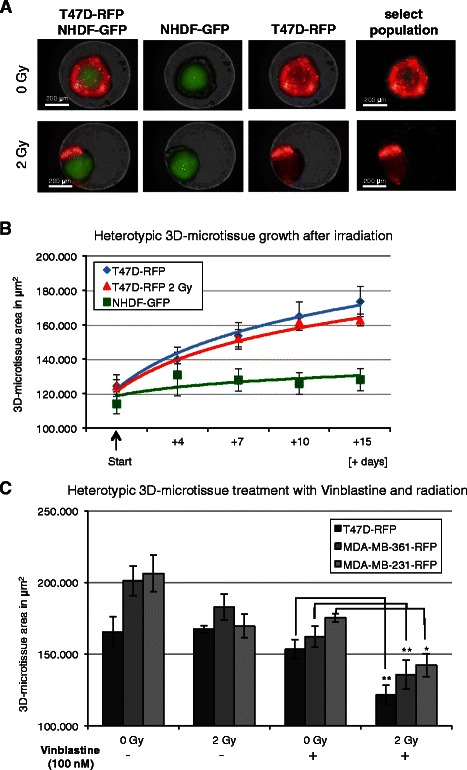
Heterotypic 3D-microtissue analysis after irradiation and concurrent Vinblastine treatment. **a** Merged and single GFP/RFP image examples of T47D-RFP/NHDF-GFP co-cultures with selected population for μm^2^ quantification after irradiation. **b** RFP and GFP plot (area in μm^2^) for T47D-RFP and NHDF-GFP heterotypic 3D-microtissues after treatment with 0 Gy and 2 Gy irradiation. **c** Co-cultures of T47D-RFP; MDA-MB-361-RFP and MDA-MB-231-RFP with NHDF-GFP growth quantification at day 11. RFP area (μm^2^) was analysed after 0 Gy and 2 Gy irradiation with concurrent 100 nM Vinblastine treatment. Data are averages ± SD, * indicate statistically (*t*-test) significant changes to corresponding control with Vinblastine treatment at 0 Gy, *p < 0.05; **p < 0.001

As mammary tumors usually include non-cancer stromal cells we investigated the contribution of fibroblasts, and potential anti-tumour bystander effects by generating heterotypic 3D-microtissues for phenotypic analysis. The inclusion of NHDF-GFP fibroblasts did not effect spheroid formation of T47D-RFP and MDA-MB-361-RFP, but allowed the previously diffusely growing MDA-MB-231-RFP cells to form 3D-microtissues (Additional file 4). These data confirm the importance of specific stromal component in heterotypic 3D-microtissue formation using different breast cancer cell lines. Growth of T47D-RFP in hetertotypic 3D-microtisues was comparable to that in monoypic cultures, whereas growth of NHDF-GFP fibroblasts was not detected (Fig. 5a and b).

As vinblastine demonstrated a radiosensitation effect in monotypic cultures we examined the effect in the 3D-heterotypic cultures. The combined treatment with vinblastine and 2 Gy radiation was an effective radiosensitizer in all three breast cancer heterotypic 3D-microtissues analyzed (Fig. 5c). NHDF-GFP fibroblasts growth after treatment was negligible (Additional file 5). Doxorubicine did not show a significant additional effect with concurrent irradiation when compared to individual substance treatment (Additional file 5), in agreement with the response of monotypic 3D-microtissues (Additional file 3). Docetaxel was not analyzed using heterotypic cultures, as radiosensitation effect was not detectable using monotypic 3D-microtissues previously.

## Discussion

Despite a number of preclinical studies indicating potential radiation sensitizing effects of candidate therapeutics, few have been tested in clinical studies. Glass and colleagues estimated that less than 10 % of phase I cancer clinical trials between 2001 and 2009 combined chemical and radiation therapy [21]. This is unusual, given that therapy with multiple drugs is common practice and that combination of chemo- and radiation therapy are predicted to be additive or synergistic [22]. One reason for the poor uptake of combined therapy in clinical trials may be the often contradictory results of preclinical and clinical models [23–27]. Very few studies have attempted to incorporate biological endpoint analysis and therefore improved mechanistic understanding between results from clinical studies and experimental approaches is needed. Traditional clonogenic survival and high throughput colorimetric assays are inadequate as drug screens to identify novel radiation sensitizers. A high content clonogenic survival drug screen has been developed recently [28], but including three-dimensional assays for drug screens could tremendously accelerate preclinical testing in the future.

The hanging drop system, the oldest cell culture technique of all, has undergone a recent rebirth [12, 29] showing great potential for making cancer screening assays more predictive and informative [5]. Therefore, 3D-microtissue technology has been adapted to create a high-throughput screen capable of following changes in cell growth in real time for up to 20 days after treatment. Three different mammary tumor cell lines were analysed in the capacity to form 3D-microtissues, confirming previously published data that not all mammary cell lines are able to self-aggregate and form spheroids [30]. Consistently cells effective in spheroid formation (T47D and MDA-MB-361) were analysed in growth delay after radiation confirming results from colony formation assays published before [17]. Furthermore, we examined ten established chemotherapeutic drugs to determine if any are capable of sensitizing a radiation-resistant mammary cancer cell line to a single 2 Gy dose of radiation. We demonstrate that the combined treatment of radiation and chemotherapeutics can be followed in real time and robustly quantified by using high content imaging platform settings and standard fluorescent area field determination per well containing single spheroids varying 10 % in size at start point of analysis (day 0).

From analysed cytostatica, taxanes such as paclitaxel and docetaxel, and vinca alkaloids such as vincristine and vinblastine, have been widely used for the treatment of a variety of tumors including breast cancer [31–33], whereas docetaxel is established as one of the most active agents against metastatic breast cancer [34].

In our analysis vinblastine emerged as the most potent radiation sensitizing agent using monotypic 3D-microtissues, suggesting that this agent can be effective when used in combined radiation and chemotherapy treatment [35]. In heterotypic cultures the combined treatment of vinblastine plus radiation was even more effective using vinblastine and indicating a cooperative bystander effect of tumour stroma in the sensitization. The serum concentration of vinblastine during cancer therapy is estimated to reach 10–400 nM a few hours after application [36, 37]. Furthermore, it is reported that vinblastine accumulates in some tissues as spleen, thyroid, large and small intestine to even higher levels, suggesting that the maximum concentration of vinblastine is in the range of 0.06–28 μM in some organs. Therefore it is conceivable that the concentration range (0.1–10 μM) we analyzed in this study is physiologically achievable. A potentiating effect of radiation with concurrent vinblastine treatment was confirmed with monotypic and heterotypic 3D-microtissue assays (Fig. 4 and Fig. 5).

The lack of an additive effect between docetaxel and radiation is in good agreement with reported lack of cooperation between paclitaxel and radiation [26, 27, 38, 39]. Despite doxorubicine having excellent anti-tumor activity no additive effect with radiation was detected in our system. This agrees with the relatively low therapeutic index of doxorubicine in metastatic breast cancer patients [40].

## Conclusions

3D-microtissue screening platforms for phenotypic drug characterisation, such as the one presented here, can accelerate the timeline for drug discovery initiatives. We have validated the screen by comparing radiation sensitizing effects of known chemotherapeutic agents. In both monotypic and heterotypic models the concurrent treatment of vinblastine and radiation proved more effective inhibitors of mammary cancer cell growth. The effective concentration range of both vinblastine and radiation are within the range used in treatment, suggesting the 3D model will offer a highly relevant screen for novel compounds.

## Additional files

Additional file 1: **Cell number correlation with spheroid growth formation.** (A) GFP area (μm^2^) settings for high content imaging platform analysis using monotypic 3D-microtissues, (B) GFP plot (area in μm^2^) quantification of spheroid growth compared to the cell number count per 3D-microtissue at indicated time points. (C) 3D-microtissue growth analysis after radiation (GFP plot in μm^2^) – area quantification of spheroid growth delay after irradiation at indicated time points up to 20 days with constant time scale and different radiation doses. Data are averages ± SD (*n* = 3). Major changes in growth delay were detected between day 3 and day 13 or 15 after starting point of treatment, therefore in all subsequent Figures this time points were used for presentation.
Additional file 2: **3D-microtissue growth analysis after treatment with chemotherapeutics.** GFP plot (area in μm^2^) of spheroid growth delay after (A) 5-FU, Cycloheximide, Etoposide, Actinomycin D, (B) Staurosporine, 6-TG, and Hydrocortisone (10 μM) treatment and quantification at indicated time points (data are averages ± SD, *n* = 3).
Additional file 3: **3D-microtissue growth delay quantification after Docetaxel, Doxorubicine and 5-FU treatment with irradiation.** GFP plot (area in μm^2^) for control T47D 3D-microtissue and after treatment with 0 Gy and 2 Gy irradiation using (A) 10 μM Docetaxel, (B) 300 nM Docetaxel, (C) 10 μM Doxorubicine, (D) 300 nM Doxorubicine, (E) 10 μM 5-FU and (F) 300 nM 5-FU at indicated time points. Data are averages ± SD, *n* = 4.
Additional file 4: **Phenotypic growth analysis of heterotypic 3D-microtissues.** Using NHDF/GFP and RFP marked breast cancer cells (A) T47D, (B) MDA-MB-361 and (C) MDA-MB-231 at day 1, day 6 and day 11 after 0 Gy (sham) or 2 Gy irradiation. Day 1 confirms 3D-microtissue assembling of heterotypic spheroids using three different tumour cell lines and growth efficiency following transfer to receiver plates up to 11 days.
Additional file 5: **Heterotypic 3D-microtissue analysis (11 days) after irradiation and concurrent Vinblastine treatment.** Co-cultures (11 days) after 0 Gy and 2 Gy irradiation and concurrent 100 nM Vinblastine or 100 nM Doxorubicine treatment using (A) T47D-RFP/NHDF-GFP, (B) MDA-MB-361-RFP/NHDF-GFP and (C) MDA-MB-231-RFP/NHDF-GFP heterotypic microtissues.

## Abbreviations

HTS: High throughput screen
HCS: High content screen
2D: Two dimensional
3D: Three dimensional
GFP: Green fluorescence protein
RFP: Red fluorescence protein
pGP: Plasmid GreenPuro
NHDF: Normal human dermal fibroblasts
DMSO: Dimethylsulfoxid
DMEM: Dulbecco’s modified eagle medium
RPMI: Roswell park memorial institute
