# Supplementary figures and images for: A 3D-microtissue-based phenotypic screening of radiation resistant tumor cells with synchronized chemotherapeutic treatment
Source: BMC Cancer. 2015 Jun 10;15:466. doi: 10.1186/s12885-015-1481-9 (PMC4460881; doi:10.1186/s12885-015-1481-9)

**A**

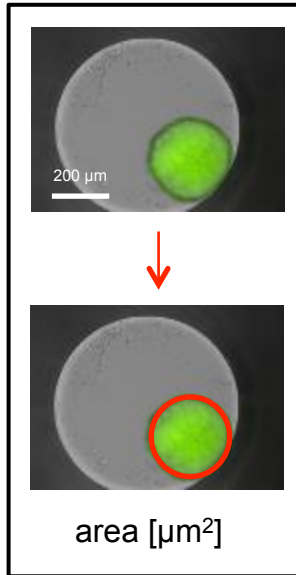

**B**

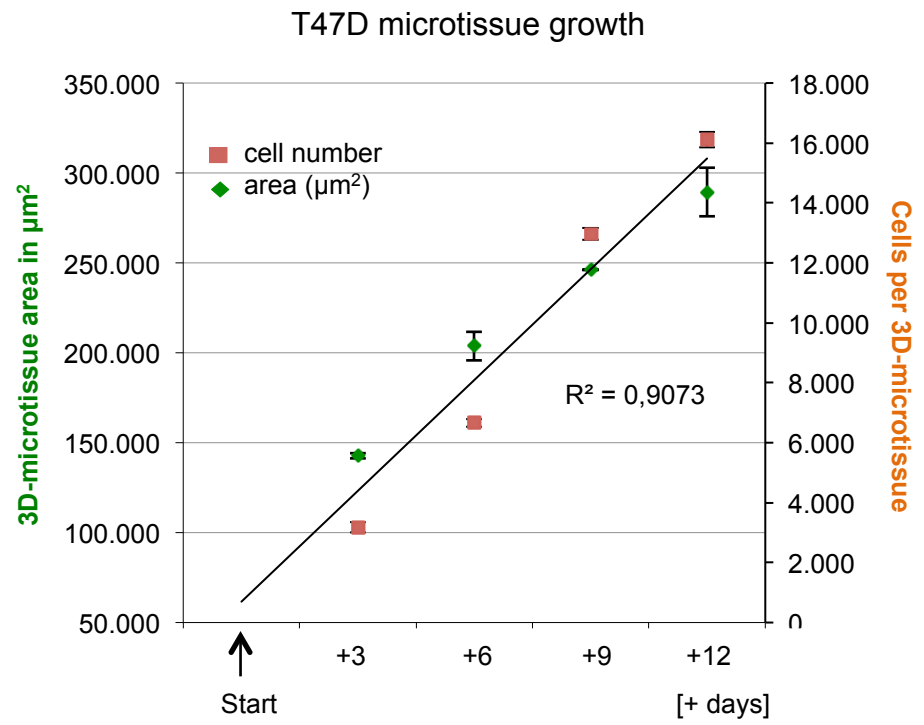

**C**

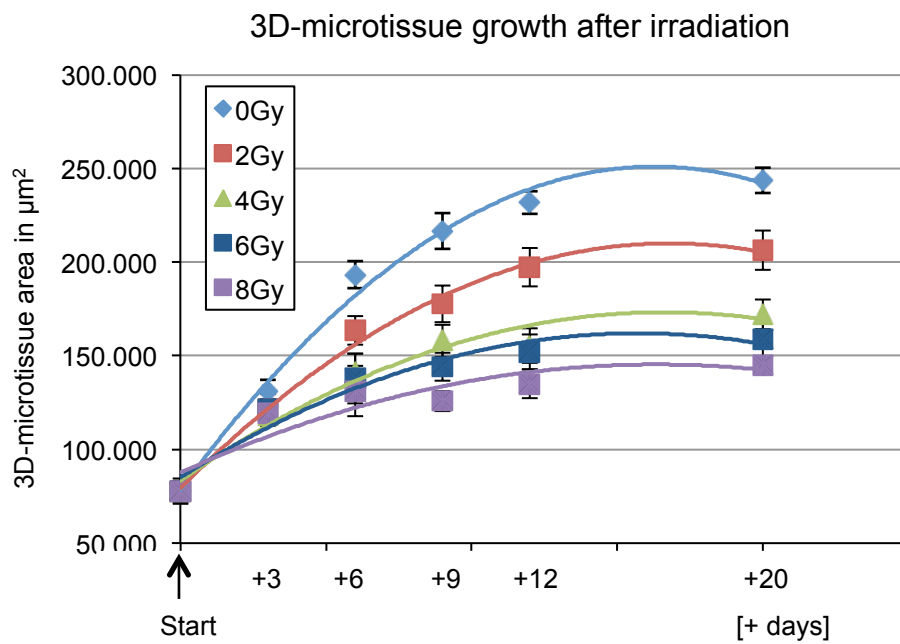

Supplement: Additional file 1: — Cell number correlation with spheroid growth formation. (A) GFP area (μm2) settings for high content imaging platform analysis using monotypic 3D-microtissues, (B) GFP plot (area in μm2) quantification of spheroid growth compared to the cell number count per 3D-microtissue at indicated time points. (C) 3D-microtissue growth analysis after radiation (GFP plot in μm2) – area quantification of spheroid growth delay after irradiation at indicated time points up to 20 days with constant time scale and different radiation doses. Data are averages ± SD (n = 3). Major changes in growth delay were detected between day 3 and day 13 or 15 after starting point of treatment, therefore in all subsequent Figures this time points were used for presentation. [file 12885_2015_1481_MOESM1_ESM.pdf]

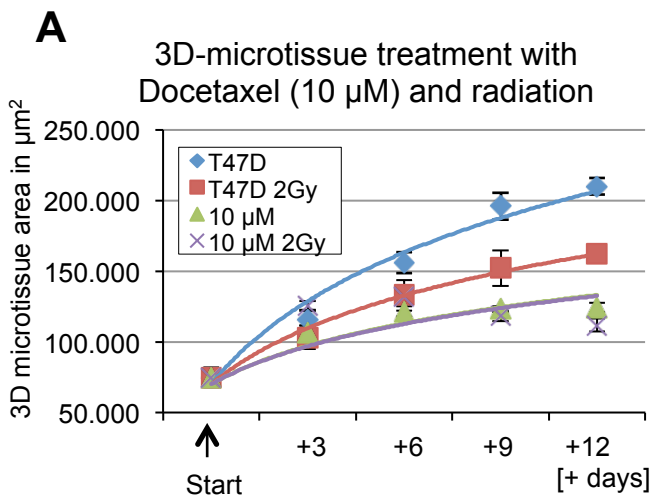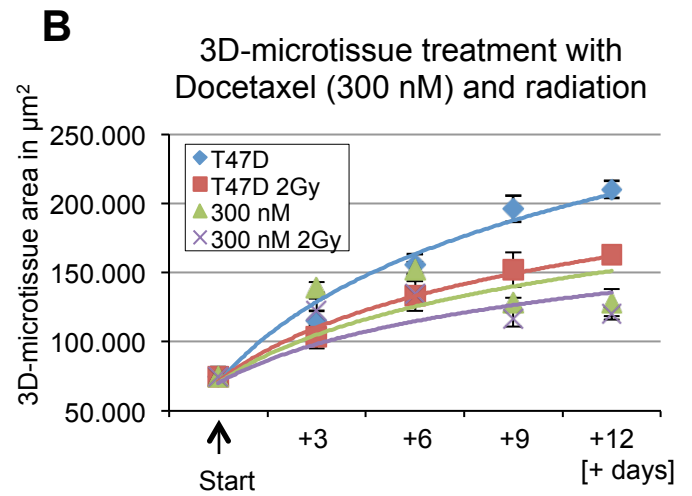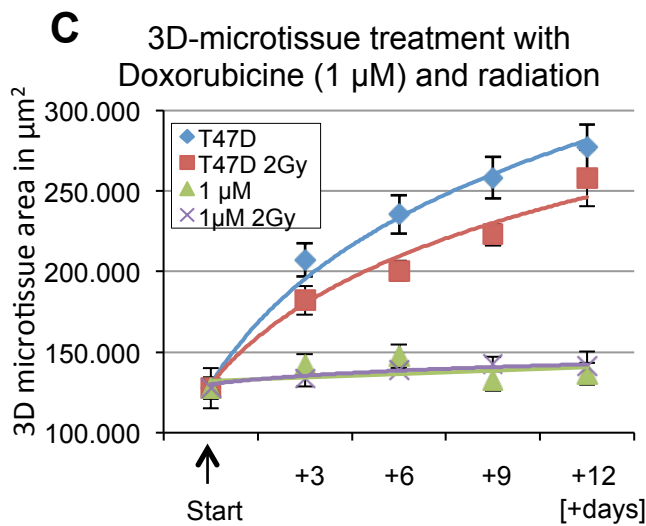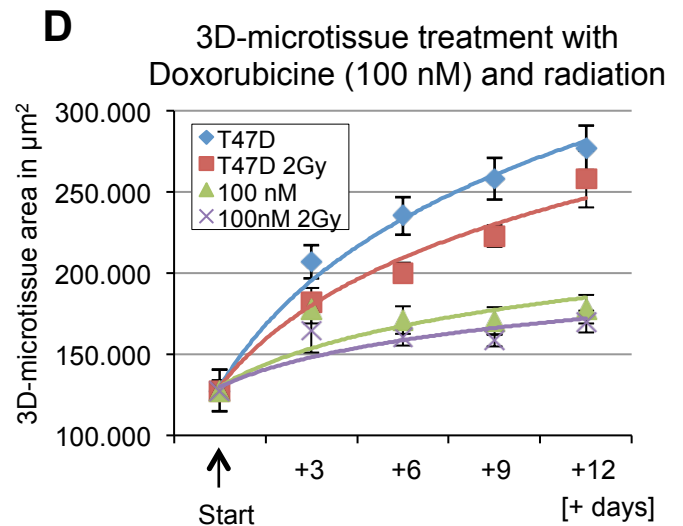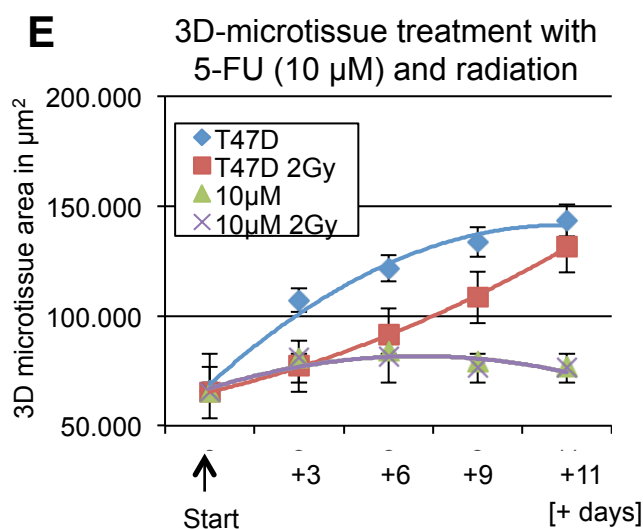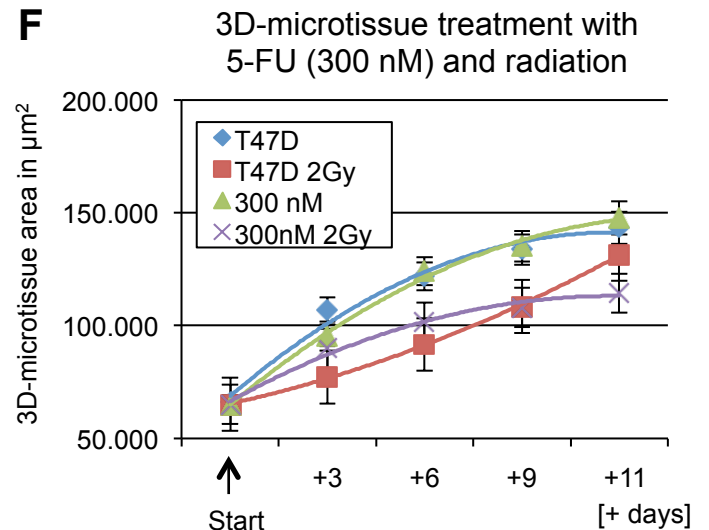

Supplement: Additional file 3: — 3D-microtissue growth delay quantification after Docetaxel, Doxorubicine and 5-FU treatment with irradiation. GFP plot (area in μm2) for control T47D 3D-microtissue and after treatment with 0 Gy and 2 Gy irradiation using (A) 10 μM Docetaxel, (B) 300 nM Docetaxel, (C) 10 μM Doxorubicine, (D) 300 nM Doxorubicine, (E) 10 μM 5-FU and (F) 300 nM 5-FU at indicated time points. Data are averages ± SD, n = 4. [file 12885_2015_1481_MOESM3_ESM.pdf]

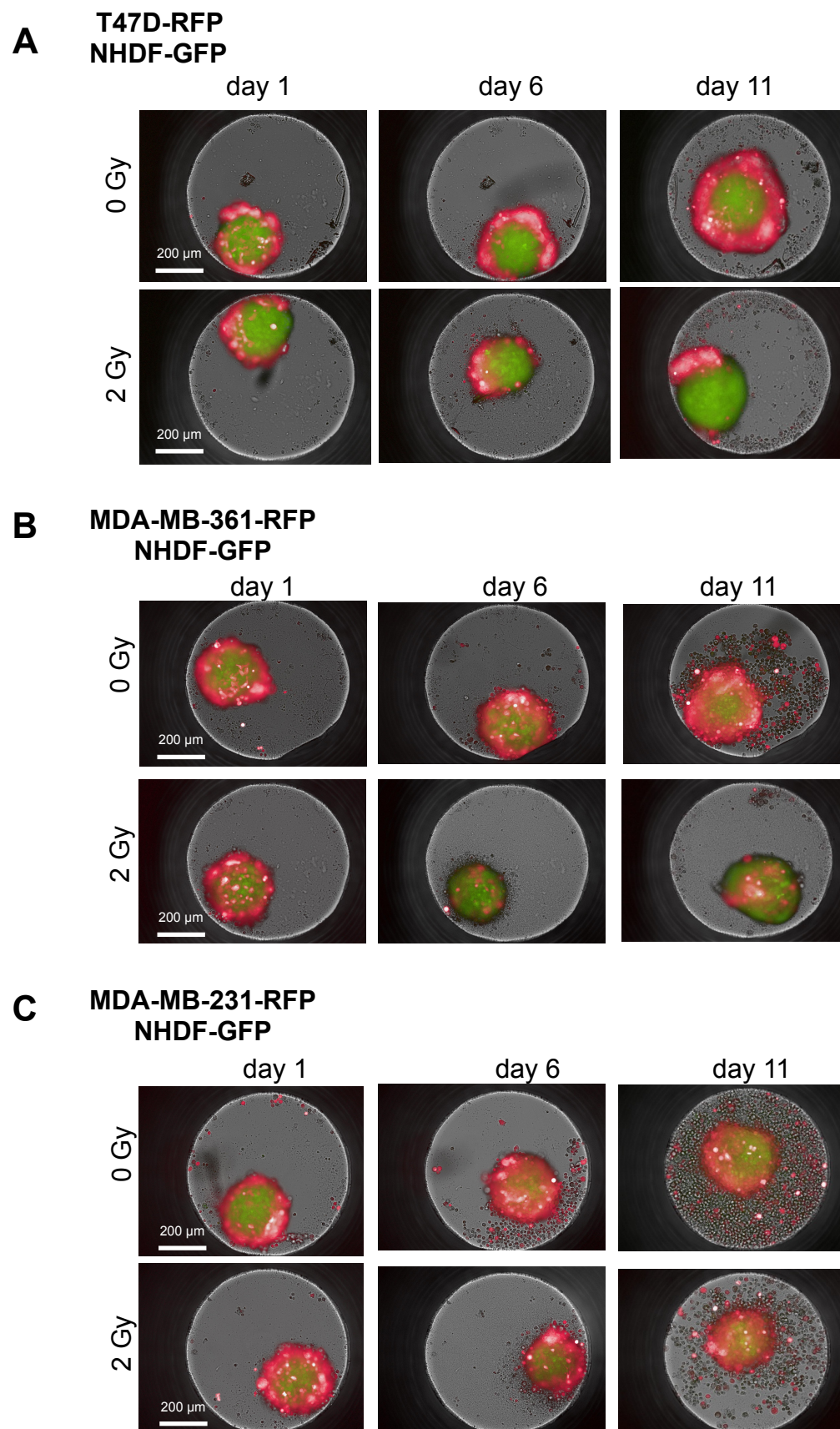

Supplement: Additional file 4: — Phenotypic growth analysis of heterotypic 3D-microtissues. Using NHDF/GFP and RFP marked breast cancer cells (A) T47D, (B) MDA-MB-361 and (C) MDA-MB-231 at day 1, day 6 and day 11 after 0 Gy (sham) or 2 Gy irradiation. Day 1 confirms 3D-microtissue assembling of heterotypic spheroids using three different tumour cell lines and growth efficiency following transfer to receiver plates up to 11 days. [file 12885_2015_1481_MOESM4_ESM.pdf]

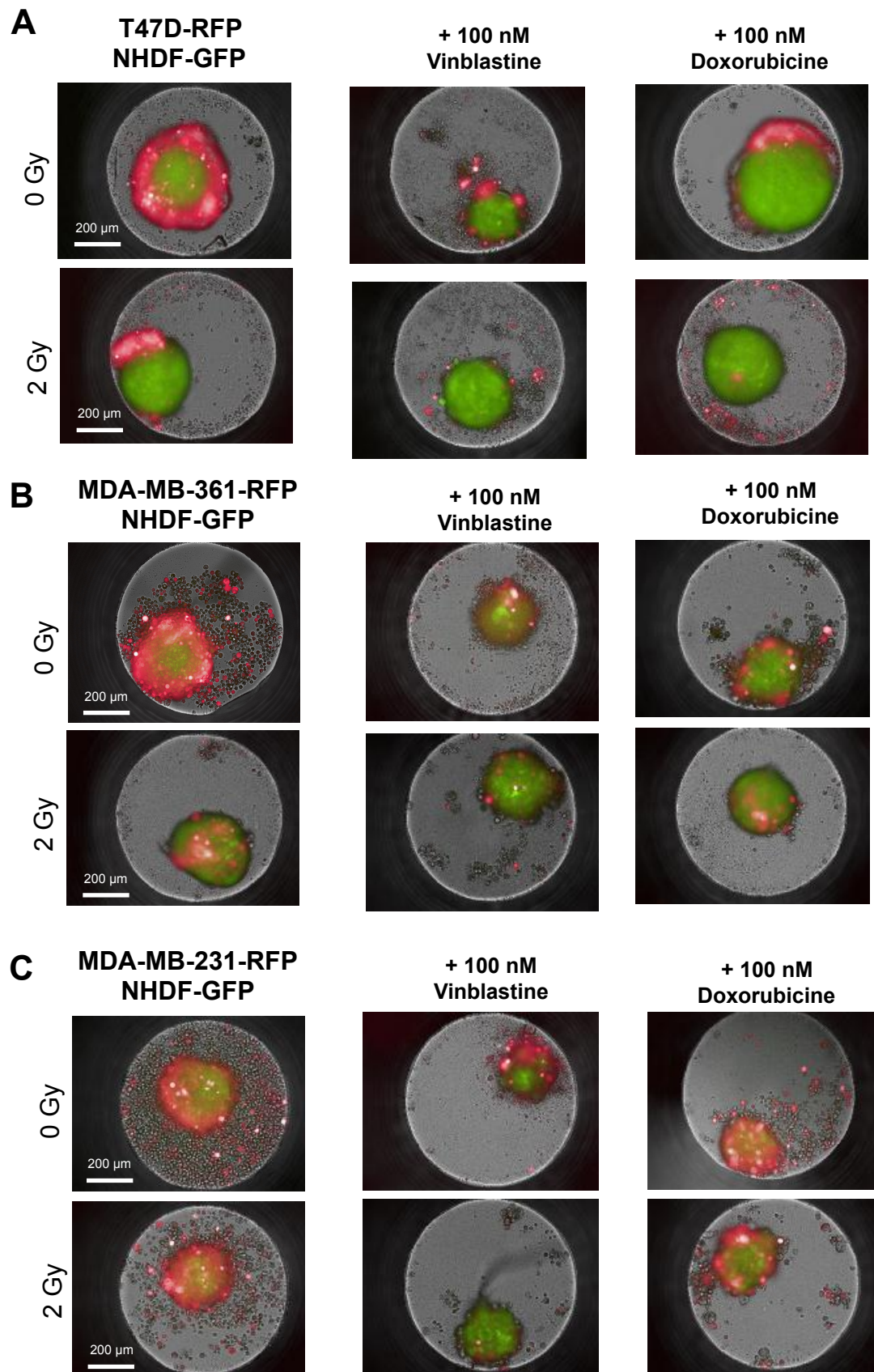

Supplement: Additional file 5: — Heterotypic 3D-microtissue analysis (11 days) after irradiation and concurrent Vinblastine treatment. Co-cultures (11 days) after 0 Gy and 2 Gy irradiation and concurrent 100 nM Vinblastine or 100 nM Doxorubicine treatment using (A) T47D-RFP/NHDF-GFP, (B) MDA-MB-361-RFP/NHDF-GFP and (C) MDA-MB-231-RFP/NHDF-GFP heterotypic microtissues. [file 12885_2015_1481_MOESM5_ESM.pdf]
