# Supplementary material for: A 3D-microtissue-based phenotypic screening of radiation resistant tumor cells with synchronized chemotherapeutic treatment
Source: BMC Cancer. 2015 Jun 10;15:466. doi: 10.1186/s12885-015-1481-9 (PMC4460881; doi:10.1186/s12885-015-1481-9)

Additional File 2

A

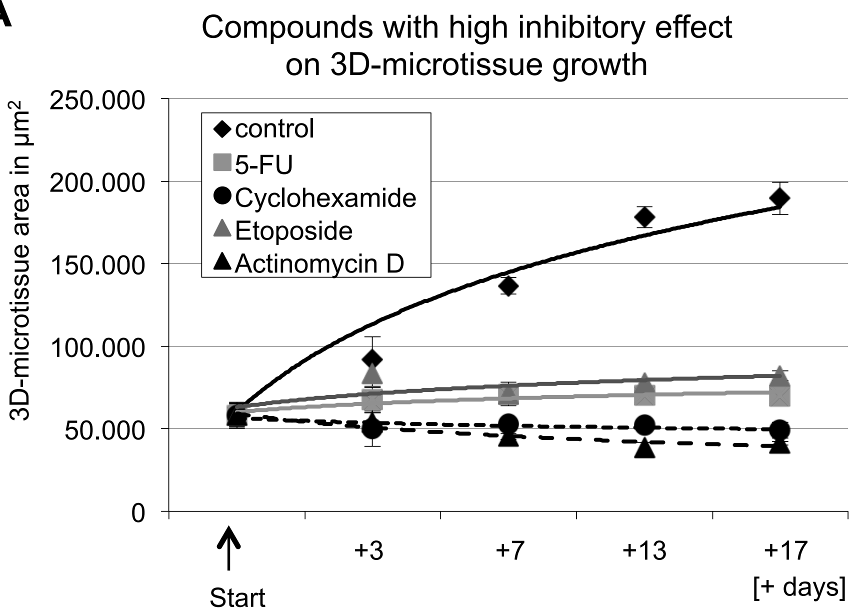

B

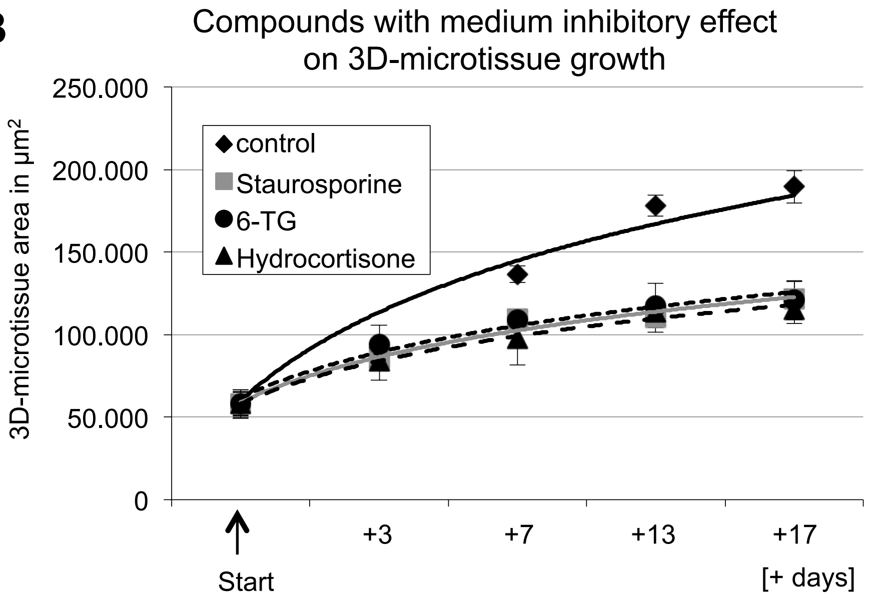

Supplement: Additional file 2: — 3D-microtissue growth analysis after treatment with chemotherapeutics. GFP plot (area in μm2) of spheroid growth delay after (A) 5-FU, Cycloheximide, Etoposide, Actinomycin D, (B) Staurosporine, 6-TG, and Hydrocortisone (10 μM) treatment and quantification at indicated time points (data are averages ± SD, n = 3). [file 12885_2015_1481_MOESM2_ESM.pdf]
